# The Method and Statistics of Suicide

**Published:** 1859-04-01

**Authors:** 


### Art. III.—THE METHOD AND STATISTICS OF SUICIDE.

It was formerly customary in this country to regard the northern portion of a churchyard as unhallowed, and to bury in it the bodies of suicides, of the executed and excommunicated, and of unbaptised infants. The more fortunate dead were interred in the southern, eastern, or western portions of the burial-ground, and headstones, or more pretentious monuments, and simple grave-mounds, freshly heaped up from year to year by the careful hands of relatives or of the sexton, marked the position of each grave; but in the northern portion of the burial-ground, the grave-mounds, disregarded, were quickly hidden amidst a rank growth of weeds, and could scarcely be distinguished if sought for, or wasting beneath the wind and the rain, they were early destroyed altogether. At any time we might readily count the graves of the fortunate dead, but few traces of the graves of the unfortunate dead would ever be found.

Much in the same fashion as the bodies of suicides were once dealt with in churchyards have the statistics of suicides been treated in the mortality records of the kingdom. Buried without distinction, within the class of deaths from external causes, these records have been hidden from the sight of the observer, except at rare and irregular intervals, when (thanks to Dr. Farr) the Registrar-General has turned aside from the well-tended figures of the legitimately dead, and brought to light those which tell of the illegitimately dead. A recent instance of this kind is to be found in the last (the 19th) Annual Report of the Registrar-General. This report contains a tabular account of the suicides which have been committed in England and Wales during the five years 1852-56, the age and the sex of the individuals who have destroyed themselves, and the mode in which the destruction was effected, being shown. According to the Registrar-General's Tables, 1045 suicides were committed in 1852; 1031 in 1853; 1081 in 1854; 1076 in 1855; and 1182 in 1856; making a total number of 5415 suicides during the five years.

The number of suicides in the mortality returns made to the Registrar-General is, according to Dr. Farr, probably less by one-tenth than the number actually ascertained to have occurred.

In 1856, the suicides noted in the registers amounted to 1182, but the coroners' returns for that year, contained in Mr. Redgrave's Tables (*Judicial Statistics*, p. 11), make the number 1314, from which, however, Dr. Farr remarks, "a few should be deducted for the duplicate return." The difference between the number of suicides returned by the coroners and the number occurring in the registers of mortality, may be owing to obscurities in the verdicts.

Of the individuals who committed suicide during the period included in the Registrar-General's Tables, 3886 were males, and 1529 females, making an average annual mortality from this mode of death of 85·1 of the former sex, and 32·5 of the latter, in every 1,000,000 individuals living from ten years and upwards of each sex respectively.

In both sexes, suicide first occurs between the 10th and 15th years of age, and from this period the gross number of suicides in each sex increases until the decennium 45—55, when a maximum is reached; after which the number steadily declines until the decennium 85—95. Subsequent to the 95th year no suicides are recorded.

If the proportion of suicides be calculated upon every 1,000,000 individuals living of each sex at different periods of life from the age of ten years, the maximum number of suicides is found to have occurred among males within the decennium 55-65; and among females, within the decennium 65-75. In the male sex the decrement of the mortality from suicide was more gradual than the increment, the number of suicides in the three decenniums succeeding the maximum, being considerably in excess of the number occurring in the three decenniums immediately preceding it. In the female sex, the proportion of suicides which occurred between the 45th and 55th years (83·6) differed but slightly from the maximum (84·0) between the 65th and 75th years, and in the intermediate decennium, 55-65, the proportion was 80·2. The decrement of the number of suicides was, moreover, less regular than among males—the number occurring in the decennium immediately succeeding the maximum falling to 43·8, while in the terminal decennium 85-95, the proportion increased to 50·9.

It would seem then, from these returns, that the greatest tendency to suicide, in this country, is manifested in the male sex from the 55th to the 65th year, in the female sex from the 65th to the 75th year, and that in both sexes the tendency to suicide is greater during middle age and the decline of life than during the earlier periods of life.

The returns of suicides for 1838-39, contained in the Registrar-General's Third Annual Report, also indicate that the tendency to suicide is greatest in the decline of life; the figures showing that, in proportion to every 100,000 individuals living at different ages, of both sexes, the maximum number of suicides occurred in the decennium 50-60, and that the proportion occurring in the three decenniums subsequent to the maximum exceeded that occurring in the three preceding it.

The following table shows the actual number of the suicides which happened at different periods of life, in each sex, during the years 1852-56, and the proportionate number to every 1,000,000 individuals living, of both the one sex and the other, in the same period.

DEATHS *at different Ages returned as having occurred from Suicide, in England, during the Five Years* 1852-56.

| AGES AT DEATH. | | | | | | | | | | | | |
|---|---|---|---|---|---|---|---|---|---|---|---|---|
| MALES. | | | | | | | | | | | | |
| All Ages. | 5– | 10– | 15– | 25– | 35– | 45– | 55– | 65– | 75– | 85– | 95 & upwds. | ? |
| 3886 | — | 19 | 348 | 547 | 726 | 910 | 778 | 410 | 127 | 9 | — | 12 |
| Deaths to 1,000,000 Living at the different periods of Life. | | | | | | | | | | | | |
| 85·1 | — | 3·8 | 40·1 | 80·0 | 138·4 | 240·0 | 311·1 | 295·6 | 252·4 | 136·2 | — | — |
| FEMALES. | | | | | | | | | | | | |
| All Ages. | 5– | 10– | 15– | 25– | 35– | 45– | 55– | 65– | 75– | 85– | 95 & upwds. | ? |
| 1529 | — | 14 | 273 | 244 | 272 | 336 | 219 | 135 | 28 | 5 | — | 3 |
| Deaths to 1,000,000 Living at the different periods of Life. | | | | | | | | | | | | |
| 32·3 | — | 2·8 | 30·2 | 33·3 | 49·3 | 83·6 | 80·2 | 84·0 | 43·8 | 50·9 | — | — |

The most interesting portion of the Registrar-General's Tables, perhaps, consists in the curious and suggestive information they contain upon the method of suicide. The returns made under this head, although confessedly imperfect, are still the most comprehensive that have yet been published in reference to this country. The modes in which suicide was effected are arranged in five classes. The *first* class contains the suicides connected with railways, the act of destruction having been effected by leaping from one of the carriages or from the engine of a train in motion, or by taking up a position in front of an approaching train and being run over. In this class ten instances, all males, are found. The *second* class contains suicides connected with mines, the individuals having cast themselves down the shafts. In this class are found 13 instances, nine males and four females, the former number being in the proportion of 0·23 per cent. of the total male suicides, and the latter number 0·26 per cent. of the total female, thus showing a slight excess among females in having recourse to this method of destruction. The *third* class contains the suicides effected by mechanical injuries, the individuals having destroyed themselves by leaping from windows, heights, or conveyances; by cutting the throat, by gun-shot wounds, or by wounds otherwise produced. In this class are found 1424 instances, 1128 males and 296 females, the former constituting 29·02 per cent. of the total male suicides and the latter 19·35 per cent. of the total female. The *fourth* class contains the suicides effected by chemical injuries, the individuals having destroyed themselves by fire or by poison. In this class are found 561 instances, 302 males and 259 females, the former constituting 7·77 per cent. of the total male suicides, the latter 16·93 of the total female. The *fifth* class contains suicides effected by suspension of the respiration, the individuals having destroyed themselves by drowning, hanging, or in some other manner causing cessation of breathing. In this class are found 3212 instances, 2285 males and 927 females, the former constituting 58·02 per cent. of the total male suicides, and the latter 60·62 per cent. of the total female. The method of suicide is not stated in 195 instances, 152 (3·91 per cent.) males, and 41 (2·81 per cent.) females.

Of the particular modes of effecting suicide among *males*, *hanging* is the commonest, this being the fashion in which death was caused in 1745 instances (44·90 per cent. of the total number), and if the number of suicides by *strangulation* (99) be added, the per centage would be raised to 47·45. Next in order of frequency is *cut-throat*, this being the mode in which life was destroyed in 810 instances (20·84 per cent.). *Drowning* stands third in the list and *poisoning* fourth, the former being the method of destruction in 434 instances (11·16 per cent.) the latter in 221 instances (5·68 per cent.).

Among *females*, *hanging* is also the most frequent method of suicide, this being the form of destruction had recourse to in 510 instances (33·35 per cent. of the total number), and the suicides by *strangulation* (28) being added, the per centage is raised to

35·18. *Drowning* has the second place in the order of frequency, this being the mode of death in 385 instances (25·17 per cent). *Poisoning* stands third in order, and *cut-throat* fourth, there being 267 instances (16·80 per cent.) of suicide among females by the former method, and 240 (15·66 per cent.) by the latter.

While, therefore, in both sexes *hanging* is the commonest method of suicide, the female has recourse to it one-third less frequently than the male. On the other hand, *drowning* is very much more common among females than males. Suicide by *cut-throat* is not so frequent by one-third among the former sex as the latter, and while the former counts only a total number of two suicides from *gun-shot wounds*, the latter counts 215. If suicides from wounds the character of which is not defined be added to those arising from gun-shot wounds, a sub-class would be formed numbering 263 males, but only 12 females. In another form of suicide from mechanical injury the number of the male sex sinks below the female, for although the total number of suicides occasioned by leaping from a window or height was in the former sex 53 and in the latter 41, the per centage upon the total number of suicides in the different sexes was 1·36 males and 2·64 females.

The number of suicides by poisoning among females not only exceeded by more than one-third those among males from the same method, but the poisons made use of by the former sex were more varied in character than those used by the latter. The female sex had recourse most frequently to *opium and its preparations* as the agents of destruction. *Laudanum* was the poison used in 29·4 per cent. of the suicides by poisoning (in which the kind of poison used is stated) among females, and if the instances in which *opium* was used be added, the per centage is raised to 36·1 per cent. Laudanum was the poison used by 28·8 per cent. of the male suicides, and the instances of suicide by opium and morphia (there being one instance only in which the last mentioned drug was made use of) being added, the per centage is raised to 34·0. But among males *prussic acid* was used in the same proportion of suicides by poison as laudanum (28·8 per cent.) and the *essential oil of almonds* was the destructive agent made use of in 14·2 per cent., these poisons together forming a per centage of 43·1. Thus prussic acid in its ordinary form, or as it exists in the essential oil of almonds, was the poison most frequently used among males, opium and its preparations holding the second place in order of frequency. *Arsenic* and *oxalic acid* hold the second place in commonness of use among the poisons used by female suicides, the per centage of each poison being the same, 18·8; but the first-named poison, arsenic, stands third in the list of frequency among males, the per centage of its use in the suicides by poison of that sex being 10·3. The per centage of suicides by prussic acid among females amounts only to 4·4, but that by the essential oil of almonds to 10·0, together forming a per centage of 14·4.

Ten different forms of poison are named as being made use of by male suicides and seventeen by female. Opium, in one form or other, was the poison made use of in 34·9 per cent. of the total number of suicides by poisoning in both sexes, and in which the kind of poison used is stated; prussic acid in its ordinary state, or as it exists in the essential oil of almonds, in 30·5 per cent.; arsenic in 14·1 per cent., and oxalic acid in 12·2 per cent.

TABLE *of Suicides by Poisoning in England during the five years* 1852-56.

| Poison used. | Males. | Females. | Poison used. | Males. | Females. |
|---|---|---|---|---|---|
| Arsenic . . . . . . | 24 | 34 | Oxalic Acid . . . . | 15 | 34 |
| Mercury . . . . . . | 2 | 4 | Sulphuric Acid . . . | 8 | 7 |
| Corrosive Sublimate . | 0 | 2 | Nitric Acid . . . . | 1 | 1 |
| Opium . . . . . . . | 11 | 12 | Muriatic Acid . . . | 0 | 2 |
| Morphia . . . . . . | 1 | 0 | Camphor . . . . . . | 1 | 0 |
| Laudanum . . . . . | 67 | 53 | Phosphorus . . . . | 0 | 1 |
| Nux Vomica . . . . | 1 | 1 | Improper medicine (*not stated what kind*) . . | 0 | 1 |
| Strychnia . . . . . | 1 | 1 | | | |
| Prussic Acid . . . . | 67 | 8 | *Not stated how or otherwise than by the above causes* . . . . . | 68 | 77 |
| Cyanide of Potassium . | 0 | 1 | | | |
| Essential Oil of Almonds | 33 | 18 | | | |

If the method of suicide be examined in reference to the age at which the deed was committed, the following results are obtained: —The suicides connected with railways commence between the 15—25th years, and no instance is recorded after the 75th year, the greatest number (3) occurring between the 45—55th years. The suicides connected with mines commence between the 10—15th years, when one female suicide is recorded, and no instance is mentioned after the 75th year among males, and after the 55th among females. The greatest number of suicides perpetrated in this mode, contained in this class, occur between the 65—75th years among males, and between the 15—25th among females, the number of suicides from 45—55, and from 65—75, in the last-mentioned sex being equal. The suicides by mechanical injuries commence by one male suicide between the 10—15th years, and increase gradually until 35—45 among males, and 45—55 among females, and in each sex, after the maximum, the numbers decrease steadily until 85—95, after which period no case is recorded. The suicides connected with mechanical injuries commence also between the 10—15th years, two female suicides occurring within that period. Among males the number increases until the period 45—55, after which it decreases, and no case is recorded after the 85th year. Among females the maximum number occurs between the 15—25th years; decreases from 25—35; again increases within the period from 35—45, and then decreases until 75—85, after which no case is recorded. The suicides connected with asphyxia commence also in the period 10—15, and in both sexes the number increases gradually from period to period, until a maximum is reached in 45—55, after which it decreases until 85—95, no instance occurring after that period.

This is the progress of the actual number of suicides by different methods in both sexes, according to age; but if the progress be regarded in proportion to every 1,000,000 individuals living at the different periods of life indicated, the maxima of suicides by mechanical and chemical injuries and suspension of respiration do not coincide with the maxima of the total number of suicides by the methods thus classed. According to the calculation named, which shows most correctly the period of greatest tendency to this or that form of suicide, the maxima in the classes named occur at the following periods of life:—Mechanical injuries—*males*, 55—65; *females*, 65—75. Chemical injuries, both sexes, 45—55. Suspension of respiration—*males*, 55—65; *females*, 65—75.

If we examine also, according to age, particular methods of suicide calculated upon the same proportion of living, it is found that, among *males*, the number of *cut-throats* increased from 15—25 to 65—75, and then decreased; *gun-shot* wounds were in greater proportion between 15—20 than 25—35. After 35 the proportion increased until 55—65, decreased in the next decennial period, again increasing from 75—85. *Poisoning* increased from period to period until 45—55, when it reached a maximum, decreased from 55—65, increased in the next decennium, and then again decreased. *Drowning* increased gradually to a maximum in 55—65, then steadily decreased. *Hanging* increased from period to period until it reached a maximum in 55—65, then decreased to the last period of life. Among *females*, *cut-throat* increased until 45—55, decreased in 55—65, attained a maximum in 65—75, decreased in the decennium following, and again increased in 85—95. *Poisoning* increased from 10—15 to 15—25, decreased in 25—35, increased to a maximum in 45—55, decreased again in 65—75, and again increased in 75—85. *Drowning* increased from 10—15 to 15—25, decreased at a slight rate until 35—45, increased in 45—55, but varied only fractionally in the three decennial periods, 45—55,

55—65, and 65—75, the maximum being in 55—65, decreased considerably in 75—85, and again increased in the last decennial period 85—95. *Hanging* increased from 10—15 to a maximum in 65—75, then decreased rapidly throughout the remaining periods. These results may be regarded as a measure of the tendency to the different methods of suicide named at the periods of life stated.

The average annual proportion of the different methods of suicide at various ages to 1,000,000 living of each sex, during the years 1852—56, is shown in the following table:—

TABLE *showing the average Annual proportion to* 1,000,000 *living of the different methods of Suicide in England and Wales during the five years* 1852—56.

| | Sex. | All Ages. | 10- | 15- | 25- | 35- | 45- | 55- | 65- | 75- | 85- | 95 & upds. |
|---|---|---|---|---|---|---|---|---|---|---|---|---|
| Connected with Railways | M. | ·2 | — | ·1 | ·3 | ·4 | ·8 | ·4 | ·7 | — | — | — |
| | F. | — | — | — | — | — | — | — | — | — | — | — |
| Mines. Coal | M. | ·2 | — | ·1 | ·1 | — | ·5 | ·4 | 2·2 | — | — | — |
| | F. | ·1 | ·2 | ·2 | — | — | ·2 | — | — | — | — | — |
| Copper, Tin, &c. | M. | — | — | — | — | ·2 | — | — | — | — | — | — |
| | F. | — | — | — | — | — | — | — | — | — | — | — |
| Mechanical Injuries | M. | 25·7 | ·2 | 10·0 | 27·2 | 54·6 | 66·4 | 85·1 | 74·1 | 77·5 | 60·6 | — |
| | F. | 6·5 | — | 3·5 | 7·6 | 10·5 | 18·3 | 15·8 | 21·8 | 9·4 | 20·4 | — |
| Chemical Injuries | M. | 6·9 | — | 4·4 | 9·6 | 14·0 | 18·9 | 14·8 | 16·5 | ·9·9 | — | — |
| | F. | 5·6 | ·4 | 7·2 | 5·7 | 10·3 | 14·1 | 10·6 | 6·2 | 7·8 | — | — |
| Asphyxia | M. | 52·1 | 3·6 | 25·5 | 42·8 | 69·2 | 153·4 | 210·4 | 202·1 | 165·0 | 75·6 | — |
| | F. | 20·1 | 2·2 | 19·3 | 20·0 | 28·5 | 51·0 | 53·8 | 56·0 | 26·6 | 30·5 | — |
| Cut Throat | M. | 18·5 | — | 3·4 | 19·4 | 40·3 | 52·2 | 62·9 | 59·9 | 50·5 | 45·4 | — |
| | F. | 5·2 | — | 2·5 | 6·1 | 9·2 | 14·8 | 13·4 | 17·2 | 6·3 | 10·2 | — |
| Gunshot Wounds | M. | 4·9 | ·2 | 6·0 | 5·9 | 7·9 | 8·7 | 15·6 | 6·9 | 12·6 | — | — |
| | F. | — | — | — | — | ·2 | ·3 | — | — | — | — | — |
| Poisoning | M. | 6·9 | — | 4·3 | 9·5 | 14·0 | 18·9 | 14·8 | 16·5 | 9·9 | — | — |
| | F. | 5·6 | ·4 | 7·2 | 5·6 | 10·1 | 14·1 | 10·6 | 6·2 | 7·8 | — | — |
| Drowning | M. | 9·9 | ·6 | 5·8 | 11·0 | 17·4 | 26·0 | 33·7 | 29·2 | 14·7 | — | — |
| | F. | 8·4 | 1·4 | 13·5 | 10·8 | 10·1 | 15·1 | 15·3 | 15·2 | 6·3 | 10·2 | — |
| Hanging | M. | 39·8 | 2·6 | 18·3 | 30·0 | 49·8 | 120·0 | 167·7 | 159·0 | 147·2 | 75·6 | — |
| | F. | 11·1 | ·8 | 5·3 | 8·5 | 17·4 | 35·0 | 35·8 | 37·7 | 18·8 | 20·4 | — |

The foregoing statistics, although chiefly valuable as presenting data for comparison with future returns, which it is to be hoped will now be regularly forthcoming from the Registrar-General's office, afford the materials for several deductions of greater or less precision.

1. The annual average number of suicides occurring in England and Wales in every 100,000 of the population, in the five years 1852—56, was, according to the preceding statistics, 5·81; and the annual average proportion per cent. in the total amount of deaths from all causes, 0·26. The third and sixth Annual Reports of the Registrar-General contain the statistics of the mortality from suicide in England and Wales for the three years 1838—40, and the annual average mortality of those years was 6·2 per 100,000 population, or 0·28 per cent. of the deaths from all causes. The average of the five years 1852—56, whether calculated upon the population or total mortality, was, therefore, less than those of the three years 1838—40. But if a more legitimate comparison of equal periods be adopted, and the average of the three years 1854—56 compared with that of 1838—39, the results are different; for in the first mentioned three years the annual average of suicides was 7·6 per 100,000 population, or ·34 per cent. of the deaths from all causes, being in excess of the annual average of 1838—40. A period of three years is not, however, sufficient to obviate the disturbances arising from the somewhat wide variations which appear to take place within short spaces of time in the number of deaths from suicide, hence no deduction can be safely drawn from the foregoing figures except in relation to the periods to which they refer. It may perhaps be surmised, the returns for 1838—40 being the only accessible returns for England and Wales previous to those for 1852—56, and consequently containing the only trustworthy data at our disposal, that no very satisfactory grounds exist for the opinion, not unfrequently entertained, that there has been a considerable increase in the mortality from suicide, in this kingdom, of late years. This surmise is strengthened somewhat by the consideration that the returns for 1852—56 exhibit, according to Dr. Farr, an improvement in precision and correctness as compared with those of 1838—40.

It would be well to ascertain, if it were practicable, the degree in which the ascertained number of suicides approximates to the actual number occurring in the kingdom. We already know that the total number of suicides contained in the Registrar-General's returns for 1856 was probably one-tenth below the number ascertained to have occurred by a coroner's inquest, and Dr. Marc d'Espine, in a work recently published (*Essai Analytique et Critique de Statistique Mortuaire Comparée:* 1858—p. 97), arguing from the acknowledged imperfections of the Registrar-General's returns for 1840, from the abhorrence of suicide in this country, and consequent supposed tendency of the friends of suicides to hush up the matter if practicable, and from the fact that the English returns for the year stated showed a proportion of suicides five times less than the annual average of the canton of Geneva, conceives that it is infinitely more probable that the number of suicides given in the Registrar-General's returns "constitute but a fifth part of the suicides which really take place in England annually." The annual average of suicides to the total mortality from all causes in the canton of Geneva during the thirteen years 1838—47, 1853—55, was 1·21 per cent.; in England during the year 1840, 0·25 per cent. "But if," writes Dr. M. d'Espine, "about 1 per cent. of deaths from suicide is wanting, in what other classes of disease have the deaths been registered? One portion would be included among violent deaths, another would receive the names of divers affections. Let us suppose 0·40 per cent. already inscribed among violent deaths, and 0·60 per cent. scattered in the remainder of the nosological arrangement, it follows that in order to complete the per centage of the violent deaths to the total amount of deaths from all causes, it is necessary to add to it 0·60 per cent. of suicides entered fraudulently in the registers. This would raise the real proportion of violent deaths in England to 4 per cent. of the total mortality, a proportion nearly identical with ours (Geneva)."

The canton of Geneva may boast of having the most perfect mortality records in Europe. The smallness of the canton has favoured the institution of regulations respecting the registration of deaths, which enable the officers of health to verify the number of deaths and their causes. The records of death from suicide in the canton are, therefore, as perfect as it is, perhaps, possible for mortality records to be. The annual average of deaths from suicide to the mortality from all causes, during a period of 13 years was, as already stated, 1·21 per cent. In England the per centage was 0·25, in 1840; 0·28, 1838—40; 0·26, 1852—56; Prussia, 0·38 (1850—2); Bavaria, 0·175 (seven years); Belgium, 0·23 (ten years); France, 0·26 (1843; Paris, 1.40); and Sardinia, 0·04 (1827—38)—the proportion in this last-mentioned country being six times less than in France and Belgium, nine times less than in Prussia, and thirty times less than in the canton of Geneva! What is the source of the great variations observed in the preceding averages? Are they to be assigned to difference of race, of habits, or of modes of thought? Or, is the explanation to be sought for in imperfections of the mortality statistics of the different countries? The last question is the one which first demands attention, for it is necessary to ascertain the worth of our data before we proceed to reason upon them. Now, Dr. M. d'Espine asserts, from the internal evidence of the returns of the countries referred to, and from the avowals of the authorities making the returns, that they are all more imperfect than the returns for the canton of Geneva, and that they differ greatly in degree of perfection among themselves: consequently, they cannot rightly be used in comparison with the returns of the canton or with one another. Moreover, he expresses the opinion that the most probable cause of the great differences observed in the annual average of the mortality from suicide in the different countries, is the greater or less degree of imperfection of their statistics, and he remarks that—

"Good statistics, those subjected to a severe criticism of the signification and value of the figures they contain, lead us more and more to recognise that facts concerning population, even in the most trifling details, are the expression of laws that scientific investigation may determine; that the variations caused by climates and races are also appreciable, but that these variations are maintained in much straiter limits than might be supposed from the very imperfect documents which statists have still to work upon; that, lastly, when great differences are found to exist between the results afforded by two countries upon one and the same question, there is less chance of deception, if the differences are attributed to an inequality in the exactness of the methods of inquiry made use of by the two countries, than in referring them to variations in facts, which might lead us to suppose that the documents were of the same value on both sides.

"I believe, then, that I may, from the whole of the data which I have given (concerning violent deaths), draw the following general conclusion :—In the majority of European States, except in case of war or revolution, 3 to 5 per cent. of the deaths will have for primary cause an exterior accident; and in every 10 violent deaths, from 2 to 4 will be occasioned by suicide, or the proportion of suicides in every 1000 deaths will be from 6 to 15. It is solely within these limits that it is necessary to seek the variations which result from races, climates, and physical and moral dispositions of populations."—(*Op. cit.* pp. 101—2.)

It may be questioned whether this conclusion is not somewhat premature, considering the scanty amount of trustworthy data at Dr. M. d'Espine's command.

The proportion of suicides which may be contained in the list of "found dead," or which may be entered in the table of violent deaths without being distinguished, or may escape the inquest of a coroner's jury in this country, we have no means of knowing, but we can scarcely conceive that it reaches the extent supposed by M. d'Espine. If, however, we are to regard the tables contained in the Registrar-General's last report as the commencement of a systematic publication of the mortality from suicide, we feel assured that in Dr. Farr's hands the statistics will, in due time, receive the highest degree of elaboration of which they are capable, and that the measure of their imperfections will be fully set forth, so that, at least, any serious errors of deduction may be avoided.

2. In England, the number of suicides occurring at different ages increases from the decennial period, 10—15, to a maximum in 45—55, after which the number declines until 85—95, no instance being recorded subsequent to the 95th year. In the canton of Geneva the number increases to a maximum in the period 20—30 (the mortality from suicide in that period being eight times greater than in the previous one). After the maximum a gradual decrease takes place until 80—90, in which period only one instance of suicide is recorded for a space of 13 years. In Paris, according to Brierre de Boismont (*Du Suicide et de la Folie Suicide:* 1856. pp. 75—76), the maximum occurs in the period 20—30, after which there is a gradual decrease until the final period indicated, 90—91. In the departments of France the maximum is not attained until 40—50, this being, in fact, about the same period that it occurs in England, if allowance be made for certain variations in the arrangement of the decennial periods in the statistics of the two countries; and if the French data be compared with the proportion of population at different ages, it is found that the number of suicides among the aged is relatively higher than among persons who have not passed mid-life. This coincides also with the results of the English returns.

The great difference which exists between the period of life at which the largest number of suicides occurs in the canton of Geneva and in England is remarkable. While in the canton the maximum is attained at the 30th year, in England it is not reached until the 55th year. In the one country it occurs in early life; in the other, when life has begun to decline: on the one hand, when the struggle of life has been more than half fought; on the other, when it has barely commenced. How significant a comment upon the difference of social or moral character in the two countries! May it not be that with this early developed tendency to suicide, telling probably either of an imperfect development of the higher moral faculties in early life, or of the too prevalent existence of notions which foster the growth of self-destruction, there is a true excess of suicides in Geneva as compared with this country; and that the high relative figure in Dr. M. d'Espine's returns, is indicative of a truth which an improvement in the statistics of other countries may only partially modify.

In Paris, the maximum number of suicides is attained at the 30th year as in the canton of Geneva. The maximum of suicides in Paris coincides with the maximum of female but not of male suicides; the greatest number of the latter taking place in the period 30—40. The difference, however, between the periods 20—30 and 30—40 numbers only 5 among males; while among females it amounts to 89. Upon the excess of suicides in Paris, from 20 to 30 years of age, Boismont remarks: "If this result be always the same, it is necessary to conclude that young persons in the capital are more addicted to kill themselves; this disposition would then be due to *ennui* (*tædium vitæ, spleen*), so common at this age."—(*Op. cit.* p. 76.) Suicide is not a necessary consequence of *ennui*, and we must seek for the cause which determines the proclivity of the *ennuyé* in Paris to self-destruction, (supposing that *ennui*, in its gravest acceptation, exists to the extent believed by M. Boismont). Is not this to be found in the conventional notions so prevalent among the youth of that city, of the fitness and legitimacy of suicide in the more serious, nay, indeed, in almost all the hitches of life? A feather will show the direction of the wind, and a waif of popular literature may indicate the tendency of thought among Parisian adolescents. In a little *brochure,* entitled, "*Paris-Medicin,*" which has casually come under our notice while writing this article, and which forms one of a series headed "*Les Petits Paris,*" (a series devoted to the oddities and characteristics of a Paris life), is represented the struggles of a young physician, and most shrewdly are the eccentricities and *dernier ressorts* of a medical life portrayed. The hero is depicted as seeking to form a practice in Paris. He has a scanty fortune, and placing his name upon the door of his apartments, with the addition, "*consultations de midi à quatre heures,*" he awaits an influx of patients. Bright visions flit before his imagination—visions of what? Of the dress affected by the noted practitioners; of the Café de Paris; of hosts of parasitic admirers; of the Opera, *danseuses,* journalism, a carriage and pair, a grand house—in which he gives superb dinners—and, finally, of a town career so bright that an enterprising editor offers him 100,000 francs for his autobiography! But the awakening tells a different tale: no patients call upon him; his funds gradually diminish; difficulty follows difficulty; and, at length, having exhausted his means, he is compelled to vacate his rooms, and wander in the streets. Then "*à bout de patience et de résignation des idées de suicide me virent.*" Aided by a friend, however, who happily comes across him before he has time to carry his suicidal notions into effect, he tries to obtain a living by practising various forms of quackery, or as the book significantly phrases it, *specialities,* but to no purpose. "*Il fallait vivre cependant, et j'étais à bout de ressources. Les idées de suicide s'offraient de nouveau à mon esprit . . . .*" In the very nick of time, the medical secretaryship to a clairvoyant is offered to him, and accepted. This is followed in succession by practice at five sous a visit, gratuitous consultations (playing into the hand of another physician), and finally, book-making. After a bitter disappointment in his literary efforts, he becomes ill, and is sent to a hospital. Recovering, he is again cast upon his own exertions for a livelihood; but although he is impoverished, and sees no prospect of success before him, he indignantly refuses a fee offered to him by a professional robber, whose brother he had aided, and his conscience pricks him that he had not denounced the malefactor to justice—"*J'ai peut-être commis un acte de coupable faiblesse.*" Then seeing that even homœopathy held out no hope to him, and that the globule had lost nearly all its influence, again comes the *refrain*, "*Il ne me restait donc plus que le suicide. Je délibérais donc sur le genre de mort que j'allais choisir.*" At this crisis, the news reached him that his aunt ("*ingrat! et je n'y songeais plus!*" had died and left him fifty thousand francs, and thenceforward life flowed smoothly.

Whether the suicidal refrain in the sketch, of which the foregoing is a description, be regarded as a satire upon a tendency of thought prevalent among a class of young men, or as the expression of an ordinary sentiment on the part of the author, it tells a tale equally significant.

3. The number of suicides is invariably greater among males than females. Boismont states that male suicides are two-thirds more numerous than female in Paris, and he gives statistics showing that the same proportion exists throughout France. This is, also, about the relative proportion in England. The data used by Dr. M. d'Espine give, as the proportion of males, in every hundred suicides, in each of the following countries, the accompanying figures:—Prussia, 82; Bavaria, 75; England, 68; France 76; and Sardinia, 80.

4. The subjoined table presents at one view the comparative prevalence of the different methods of suicide in England and several continental countries:—

| | England, 1852—56. | France, 1835—43. | Geneva, 13 years. | Sardinia, 1843—53. | Belgium,* 1836—39. | Saxony,* 1830—34. | Norway,* 1836—45. | Denmark,* 1835—44. |
|---|---|---|---|---|---|---|---|---|
| Hanging and Strangling . . . | 1 | 2 | 3 | 3 | 1 | 1 | 1 | 1 |
| Cut throat and wounds not by firearms . . . . . . . | 2 | 6 | 4 | 4 | — | — | — | — |
| Drowning . . . . . . . . . | 3 | 1 | 2 | 2 | 2 | 3 | 2 | 2 |
| Poisoning . . . . . . . . . | 4 | 7 | 6 | 6 | — | — | — | — |
| Gun-shot wounds . . . . . . | 5 | 3 | 1 | 1 | 3 | 2 | 3 | 3 |
| Asphyxia by Carbonic Acid Gas | — | 4 | 7 | 7 | — | — | — | — |
| Fall from an elevated place . . | — | 5 | 5 | 5 | — | — | — | — |

The foregoing figures must not be taken as absolute, as the position of the principal methods of suicide may vary in relation to each other in different periods. Thus from 1825 to 1834, *drowning* was the commonest mode of suicide in the canton of Geneva, but in the thirteen years 1838—47, 1853—55, gunshot wounds stood first in order of frequency.

The three chief methods of suicide—hanging, drowning, and

* *Traité de Géographie et de Statistique Médicales.* Par J. Ch. M. Boudin, 1857, t. ii. p. 82.

wounds by various instruments—are those over which the least control can be exercised, and which can be most readily had recourse to. Facility of access to deleterious agencies has, however, a decided influence upon the method of suicide employed, as is shown by the high position which poisoning holds in the English returns as compared with those of France and Geneva. It has often been urged upon the attention of government and the public that the almost unrestricted sale of poisons in this country facilitates the perpetration of suicide, and every addition to our statistics strengthens this opinion. The bill at present before parliament for regulating the sale of poisons will, doubtless, have some effect in diminishing the number of suicides by poison; but the provisions of the bill only interpose slight checks to the sale of the poison most commonly used by suicides. Suicides by opium and its preparations form, however, but a small item in the mischief done by the ignorant use of that drug, and it may be doubted whether it be sound policy to exempt the most familiarly known, most frequently used, and the most mischievous of all the poisonous drugs, from the more stringent restrictions of the bill.

It has been objected to the argument derived from suicides against the free sale of poisons that, if they were not attainable, the persons who now have recourse to poison for self-destruction would adopt some other method of suicide, and hence that no advantage would be gained by restriction. This objection is entirely speculative; but it is a matter of experience that the method of destruction exercises an important influence in determining the act of suicide, and that many who would have recourse to poison, shrink from more violent means of death. It is not to be forgotten, that the suicides by poison only represent a portion of the cases in which poison was used with a suicidal intention.

---

